# Real emotional experience of family members of patients transported within hospital in neurosurgical intensive care unit: A descriptive qualitative study

**DOI:** 10.1002/nop2.2151

**Published:** 2024-05-21

**Authors:** Guo Xuan, Ding Juan, Zeng Xurui, Liu Fei

**Affiliations:** ^1^ Department of Neurosurgery Jingzhou Hospital Affiliated to Yangtze University Jingzhou Hubei China; ^2^ Nursing Department Jingzhou Hospital Affiliated to Yangtze University Jingzhou Hubei China; ^3^ Medicine Department Yangtze University Jingzhou Hubei China

**Keywords:** experience, family members, intra‐hospital transport, neurosurgical intensive care unit, qualitative study

## Abstract

**Aim:**

To understand the real experience of family members of patients in neurosurgical intensive care unit (NICU) during intra‐hospital transport (IHT), explore their inner needs and provide effective intervention measures for the construction of standardized IHT plan.

**Design:**

A descriptive qualitative study.

**Methods:**

For the purposes of this study, 10 family members of IHT patients were included using a purposive sampling method. Semi‐structured in‐depth interviews were used to collect the data, Nvivo 11 software was used to organize the data, and Colaizzi's 7‐step descriptive phenomenology method was used to analyse the data.

**Results:**

A total of three themes and nine subthemes were extracted, namely: Experience of emotional changes at different stages (uncertainty before transfer, complex internal activity during transit, ambivalence after transfer); Perception of problems in IHT (poor doctor–patient communication, weak awareness of risk assessment, deficiencies in the transfer procedure); Consciousness of the real needs (emotional respect and closeness, stay informed of the progression of the disease, greater social support).

**Conclusion:**

Family members of patients in the NICU have complex internal experiences and multiple support needs during IHT, reflecting the need for further standardization of the transport process. In the future, we should improve the mode of safe IHT involving doctors, nurses and family members of patients, ensure the safety of patient transport, meet the social support needs of family members and improve the experience of IHT and the medical satisfaction of family members.

## 1 INTRODUCTION

Safely transporting patients and reducing the incidence of adverse events in the process of transportation are one of the core contents of hospital safety management (Zhang et al., 2019). As a hot topic in the research of domestic and foreign scholars in recent years (Ding et al., 2021; Lin et al., 2021; Zhang et al., 2019), it is obviously pointed out in the activity plan of the ‘High Quality Nursing Service Demonstration Project’ in 2010 issued by the General Office of the Ministry of Health of China that: improving patients' satisfaction with nursing services and providing safe nursing services. In 2015, ‘patient transport’ is ranked as one of the top 10 safety concerns for American medical institutions (Blakeman & Branson, 2013). In May 2019, the British Intensive Care Society released the 2019 edition of the guidelines for the transport of critically ill adults, the guidelines cover 19 aspects, including transfer decisions, ethical guidelines, patient and family communication, transport mode, support personnel and risk assessment, transport preparation, checklists, monitoring during transport, transport safety, air transport recommendations, document transfer, insurance and compensation (Fu et al., 2021).

Being critically ill and having numerous tubes in place, patients may get worse rapidly. Any accident in the course of intra‐hospital transport (IHT) may result in a drop in SpO_2_, blood pressure fluctuations, unplanned pipeline detachment and a deepened disturbance of the patient's consciousness, even severe complications such as respiratory arrest and cardiac arrest and death occur (Bender et al., 2019; Hosmann et al., 2021; Kuchler et al., 2019) and adverse events such as insufficient oxygen, insufficient power and missing items may also occur, and the risk of transport is high (Kuchler et al., 2019; Zhang et al., 2019).

As the Institute of Medicine Committee reports Bridging the Quality Divide: A New Health System for the 21st century among the six key areas for improvement detailed in this report, patient‐centred care is defined as ‘providing care that respects and responds to individual patient preferences, needs and values, and ensure that patient values guided all clinical decisions’. Admission of Intensive Care Unit (ICU) patients is associated with a range of psychological problems in their family members, termed Family Intensive Care Unit Syndrome (FICUS) (Saeid et al., 2022). The incidence of FICUS may have different consequences, including barriers for family members in the decision‐making process (Saeid et al., 2020). Even after ICU patients are discharged from the hospital, their families may experience fresh or worsening physical, cognitive or mental impairments that may last for months to years, called post‐intensive care syndrome (PICS) or post‐intensive care syndrome‐family (PICS‐F) (Saeid et al., 2022). It is known to have a prevalence of 6%–57% (Connolly et al., 2016). Family members, as the patient's supporters and decision‐making agents, not only bear a heavy financial burden but also face great psychological stress from the patient's uncertain prognosis and threat of death. They are also in a state of crisis, which can lead to a decline in the potential coping ability and medical decision‐making power of the patient's family members and, to a considerable extent, a negative impact on the patient's treatment course and disease prognosis (Saeid et al., 2020). However, families of ICU patients often receive little attention from healthcare providers. It also seems to be a departure from patient‐centred care, in which clinical decisions made by family members can be extremely compromised (Johnson et al., 2019).

Family education programs have been shown to be useful for ICU families and should be incorporated into clinical care (Avci & Ayaz‐Alkaya, 2022). In addition, supporting families can help improve patient outcomes by enabling families to provide more effective care (Davidson et al., 2017; Gerritsen et al., 2017). Communication with family members can increase family satisfaction, improve quality of care, build trust between physicians and patients and reduce conflict (Gerritsen et al., 2017; Harlan et al., 2020). ICU medical staff should use communication skills such as active listening and empathy (Davidson et al., 2017; Gerritsen et al., 2017). Before the transfer, it is necessary to communicate with the patient's family members, inform the risk of transfer and obtain the informed consent and cooperation of the family members (Liu, Fu, & Ding, 2020; Liu, Fu, Ding, & Yan, 2020). Patients and their families should be informed about all stages of the transfer process and should be provided with appropriate written information (Fu et al., 2021). To improve the safety of IHT of critically ill patients, we should not only pay attention to the configuration of equipment and drugs, but also emphasize the construction of soft factors such as personnel, systems and communication (Hu et al., 2021). The construction of the review index for IHT of critical patients also confirms the importance of consultation and cooperation between medical staff and family members (Ding et al., 2021). Therefore, in view of the IHT of neurosurgical intensive care unit (NICU) patients, exploring the experiences of family members during transfer helped healthcare workers to pay additional attention to the issues they face, and it is particularly essential to respond positively to their intrinsic needs.

## 2 BACKGROUND

IHT refers to the movement of patients between different units of a hospital to achieve diagnostic and therapeutic goals (Murata et al., 2022). Patients in the NICU are transported within the hospital for head CT and MRI, and undergo surgery or transfer to an operating room or intervention room (Bender et al., 2019; Chaikittisilpa et al., 2017). The primary goal of admission to the NICU is to maintain the intracranial pressure and the ventilator primarily within the normal range, thereby preventing secondary bleeding and injury (Bender et al., 2019). However, increasing intracranial pressure and decreasing cerebral perfusion pressure, as well as changes in cardiopulmonary function, are common complications during IHT in NICU patients and can result in secondary brain injury (Bender et al., 2019; Hosmann et al., 2021; Kuchler et al., 2019), with an adverse event rate as high as 79.8% (Hu et al., 2021; Jia et al., 2016).

A large amount of literature, both domestic and foreign (Jia et al., 2016; Liu, Fu, & Ding, 2020; Liu, Fu, Ding, & Yan, 2020; O'Leary et al., 2018; Zeng et al., 2021; Zhang et al., 2019), shows that common risk factors for IHT adverse events include patients, transport organization, technology, personnel and collective factors. O'Leary et al. (2018) have revealed that the incidence of adverse events in IHT of critically ill patients is strongly dependent on the patient's condition, equipment, transport personnel and transport organization. Zeng et al. (2021) have proved that the risk of IHT should be controlled from the aspects of the process, transport personnel and equipment to reduce the incidence of adverse events in IHT. Thus, the key to preventing various adverse events in critically ill patients during transport lies in the implementation of correct transport decisions, predictable pre‐transport preparation, disease monitoring during transport and seamless post‐transport handover (Liu, Fu, & Ding, 2020; Liu, Fu, Ding, & Yan, 2020; Zeng et al., 2021; Zhang et al., 2019).

Family members are not only a vulnerable group at higher risk of health decline after a patient was admitted to the NICU but also act as important decision‐makers and supporters of planned care (Avci & Ayaz‐Alkaya, 2022). They are subjected to high levels of psychological stress due to adverse events during the IHT. As a result, many negative emotions are generated (Karlsson et al., 2020), not only does it damage their own psychological, physical and social relationships but it also leads to decision‐making disorders that affect patients' treatment and disease prognosis (Saeid et al., 2020, 2022). Psychological problems for the patient's family members can range from severe negative emotions on admission to the ICU to decreased quality of life over time. Studies (Saeid et al., 2020, 2022) have reported that there is a high prevalence of anxiety (70%–80%), depression (35%–70%), post‐traumatic stress disorder (57%), fatigue, sadness and fear (80%) among family members of ICU patients. One study (Saeid et al., 2020) shows that family members of ICU patients experience severe physical, psychological and social stress, and that these issues can severely affect the health of family members and lead to problems such as limited rest, sleep and physical activity. These negative emotions are bound to affect the satisfaction of patients and their families during medical treatment (Li et al., 2023). However, family satisfaction refers to the extent to which healthcare professionals meet the needs and expectations of family members and how family expectations, information and communication affect family satisfaction (Weber et al., 2021), and thus has become an important parameter reflecting ICU quality (Saeid et al., 2020).

Negative and positive experiences coexist in the family of a critically ill neurosurgical patient, and the accompanying experiences and needs at different stages of the disease exhibit dynamic changes. It is recommended that medical staff properly guide clinical decision‐making for family members, develop targeted management strategies based on their needs and explore the potential of family members from a positive perspective, which can help improve their coping ability and maintain stable mental health of family members (Lin et al., 2021). Implementation of an evidence‐based standardize transfer process can effectively reduce the incidence of adverse events in neurosurgical critical care patients and improve patient family satisfaction (Zeng et al., 2021). In order to reduce the incidence of adverse events in IHT and to provide safe and efficient medical and nursing services to patients, it is imperative to find an effective and feasible method to guide medical staff in conducting IHT of critically ill patients. Therefore, family‐centred care should be considered an important skill for NICU medical staff (Gerritsen et al., 2017). Understanding the inner world and coping styles of family members of NICU patients during visits is an issue of great concern to medical staff (Li et al., 2023).

As a special research project of the Hubei Provincial Administration of Science and Technology Innovation, this study follows a previous survey on the state of knowledge and implementation of IHT for critically ill patients by medical staff (Liu, Fu, & Ding, 2020; Liu, Fu, Ding, & Yan, 2020), a summary of the best evidence for IHT in critically ill patients (Liu, Fu, & Ding, 2020; Liu, Fu, Ding, & Yan, 2020), the formulation of evidence‐based review metrics for IHT in critically ill patients and analysis of barriers (Ding et al., 2021), the application of evidence‐based standardized process in IHT of critically ill patients in neurosurgery (Zeng et al., 2021), etc. Although safe transport of critically ill patients has now become one of the hot topics of research for scholars at home and abroad, its main focus is on emergency departments and ICU, and studies specifically on IHT of NICU patients are rare. Despite the importance and prevalence of FICUS and its significant impact on family members, little research has been done in this area. At the same time, there is a lack of focus on family members (Karlsson et al., 2020). Previous studies on the psychological problems of family members of ICU patients have mostly used quantitative designs to assess the prevalence of anxiety, depression and other problems, so the depth of data from FICUS is limited (Saeid et al., 2022). Therefore, in view of the IHT of NICU patients, this paper used qualitative research methods from the perspective of family members and strove to explore the real emotional experience of family members during the transfer, so as to provide a reference for improving the standards of neurosurgical critically ill patients' transfer.

## 3 THE STUDY

### 3.1 Design

The central question guiding this study was: What was the true emotional experience of family members of patients in NICU during IHT? This study provided families with a voice to describe their interactions with transportation healthcare providers. Qualitative descriptive methods were used to collect responses to open‐ended questions from a family perspective.

### 3.2 Method

#### 3.2.1 Setting and participants

The participants were NICU family members from a Class III general hospital (which provides medical and health services across regions, provinces, cities and the whole country, is a medical and preventive technology centre with comprehensive medical treatment, teaching and scientific research capabilities. Its main function is to provide specialized medical services and solve critical and difficult diseases) in Jingzhou City, Hubei Province, China. Participants were recruited using a targeted sampling method according to the following criteria. Inclusion criteria: (1) The patient's spouse, or blood‐related relative/the patient's main caregiver and the person responsible for medical decision‐making; (2) Participating in the whole process of IHT for ≥3 times; (3) Volunteer to participate in this study. Exclusion criteria: (1) Family members of patients who stayed in NICU for less than 24 h; (2) <18 years old; (3) Additional traumatic experiences (such as bereavement, divorce, etc.) within half a year (Lin et al., 2021); (4) Previous history of mental illness, severe cognitive impairment or psychological problems.

#### 3.2.2 Data collection

The senior researcher (DJ) was the deputy director of the nursing department of a tertiary hospital and the teaching teacher of nursing graduate students in a university. She has a master's degree and served as a nursing graduate mentor to the researchers of this study, all of whom had previous experience in qualitative research. As leader of the IHT program, DJ again trained all the researchers in qualitative research. The semi‐structured interviews were conducted between June and August 2022. Based on a search of relevant literature at home and abroad, and repeated discussions among members of the research team, a first draft of the interview outline was drawn up in consultation with two chief neurosurgeons, two specialist neurosurgery nurses and a consultant psychiatrist in neurosurgery. The interview consisted of open‐ended questions: (1) ‘Please tell me about your family's experience and feelings of IHT in NICU?’ (2) ‘Describe what impressively happened during the IHT process?’ (3) ‘What links do you think will affect the safety and timeliness of IHT of patients?’ (4) ‘From the perspective of the patient's family, do you have any comments or suggestions on the IHT process of NICU patients?’ These questions were used to support the interview process and to encourage participants to elaborate on their responses. The researchers chose the time (i.e. working hours) and agreed with the interviewees on the time and place of the interview. For this interview, NICU Talk was chosen as the location and the agreed interview time was 30–50 min. Before the interview, make sure the environment was quiet, secluded and undisturbed, and talked casually to the interviewee to lighten the atmosphere and relieved their nerves. During the interview, a part‐time nursing graduate student (GX) in a university listened attentively and recorded the whole process, and was responsible for carefully observing and recording the tone, intonation and expression of the interviewees. During the formal interview, another full‐time graduate student (LF) from the same university extracted an opinion statement that was consistent with the research phenomenon, encouraged participants to express their true and deep feelings and thoughts and finally summarized and refined common concepts from meaningful statements to form a theme. Interview transcripts were transcribed into text within 24 h of the end of each interview to collate and refine research notes, general information about the research topic and non‐verbal information. All interviews were audio‐recorded and transcripts were written in Chinese. A total of 14 interviewers were invited for this study and 12 ended up being interviewed. At the end of the interview, participants received a gift worth RMB 30 as a reward for their time.

#### 3.2.3 Data analysis

Our researchers were graduate students who have systematically studied and trained in the theory and practice of qualitative research, qualitative research methods and interview techniques. After the interview, a part‐time nursing graduate student (ZXR) transcribed the records into word‐by‐word text data within 24 h and gave feedback to the participants via WeChat (a Chinese mobile phone/web application used for messaging or communication) to verify the authenticity of the content. Finally, all data will be fed into the qualitative research data management software Nvivo 11 for analysis. Two researchers (GX and LF) analysed and coded the data independently. Descriptive phenomenological methods were used, and the data were analysed according to the seven‐step method of Colaizzi (Sanders, 2003), this approach focused on integrating the feelings of research participants and added steps for research participants to confirm and evaluate the conclusions of the researchers' analysis. The detailed procedures were as follows: (1) Within 24 h after the interview, the interview data were read independently by two researchers. (2) Researchers considered and repeatedly analysed the data to identify and extract recurring ideas or meaningful expressions. (3) Coding meaningful statements. (4) The codes were pooled to form the initial theme. (5) Describe the subject in detail and aptly in relation to the actual situation of the research object. (6) Research group discussion to sublimate the theme concept. (7) To verify the results of data analysis. Doing so improved the validity of the qualitative study structure through the study participants and allowed new information that emerged during the validation process to be integrated into the detailed description. The sample size was saturated when the information was repeated and no new topics were presented. When we recruited 10 family members involved in IHT in the NICU, the information reached saturation and we stopped data collection, finding no new topics. The general information of the 10 family members is given in Table 1.

**TABLE 1:** Basic information of 10 family members.

No.	Gender	Age (years)	Education level	Occupation	Relationship with patients	Patient disease diagnosis	Patient age (age)	Patient NICU length of stay (day)	Number of IHTs attended
1	Male	36	Master's	Doctor	Son	Hypertensive cerebral haemorrhage	65	10	4
2	Female	38	Junior College	Unemployed	Spouse	Severe craniocerebral injury	39	7	3
3	Male	40	Junior high school	Farmers	Son	Epilepsy, bleeding in the brain	68	12	6
4	Male	47	High school	Businessman	Brother	Brain tumours	49	15	7
5	Female	33	Undergraduate	Teachers	Daughter	Glioma	56	29	9
6	Male	30	High school	Workers	Grandson	Multiple trauma	77	15	6
7	Male	57	Elementary school	Retirement	Son	Arteriovenous malformation, bleeding from the brain stem	34	22	8
8	Female	48	Elementary school	Farmers	Spouse	Cerebral haemangioma	50	10	4
9	Female	35	Master's	Civil service	Daughter	Bleeding from the brain stem	58	20	7
10	Male	58	Junior high school	Workers	Father	Cerebral haemangioma	36	17	6

Abbreviations: IHT, intra‐hospital transport; NICU, neurosurgical intensive care unit.

### 3.3 Ethics statement

Before the interview, the researcher informed the family of the purpose and content of the interview, explained to the family that the patient's real name would be replaced by the patient's medical number for protection and promised confidentiality throughout the process. After consent was obtained, the interviews were recorded live. All respondents signed an informed consent form. In accordance with the requirements of the Helsinki Declaration. Respondents were numbered from 1 to 10, not by name, to protect their privacy. The study was approved by the ethics review board of a university‐affiliated hospital, the ethics approval number was 2023‐026‐01.

### 3.4 Validity and rigour

Triangulation combined at least two or more theoretical perspectives, methodologies and data sources, investigators or data analysis methods. The purpose of using triangulations was to reduce, negate or balance the inadequacy of a single strategy, thereby increasing the power to interpret the results (Thurmond, 2001). When more than one type of triangulation was used, for example, two or more data sources together with two or more investigators, the resulting complex triangulation was referred to as multiple triangulation. In this study, the data source triangulation (data were collected at multiple time points according to the different characteristics of the objects studied), investigator triangulation (two researchers analysed the same data), methodological triangulation (various methods of data collection, such as conference notes, observation, recording) and data‐analysis triangulation (continuous and repeated data analysis, with results continuously compared to the original data) were adopted. We classified patients by different characteristics in terms of age, sex and education. Two investigators (GX and LF) repeatedly read, analysed and coded the same textual data, wrote reflective diaries and continuously compared the results with the original data. To improve efficiency, all interviews were transcribed verbatim without adding any precise standard concepts, and data coding was done independently by three researchers (GX, LF and ZXR). In coding, researchers exchanged opinions on discrepancies between codes and invited a third‐party member to arbitrate and reach a consensus. After the data analysis, the team performed a correction to ensure the accuracy of the analysis. The credibility of the results was established through membership verification and peer reporting. Transitivity was achieved by providing a detailed description of the learning process. In terms of reliability and confirmability, we conducted an audit trail of the interview data and the research process and checked the consistency of the results. Finally, the leader (DJ) of the research group organized the discussion and analysis of the different views of the researchers to form the final theme, so that the analysis and interpretation were more reasonable and logical, and strove to improve the credibility of the data.

## 4 RESULTS

**FIGURE 1:**
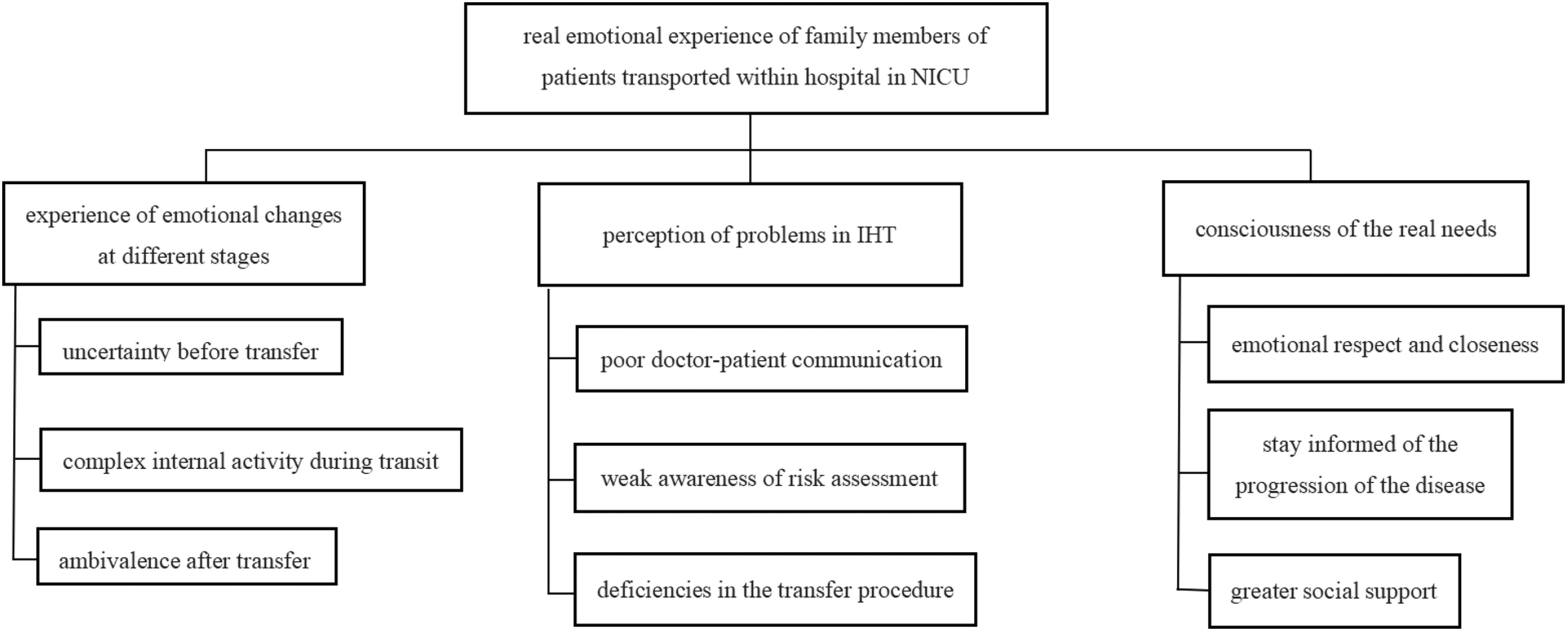
Themes and sub‐themes.

After repeated reading of the interview material, the study group discussion refined three themes and nine sub‐themes to form Figure 1.

### 4.1 Theme 1: Experience of emotional changes at different stages

This theme mainly contained the following three sub‐themes: uncertainty before transfer, complex internal activity during transit and ambivalence after transfer.

#### 4.1.1 Sub‐theme 1: Uncertainty before transfer

During this time, thoughts of how to practically and safely conduct patient transport can cause a combination of concern and suspicion among family members. Transportation was often unexpected due to the variable condition of patients in the NICU. When they were told they needed to be transported, family members expressed confusion about whether they were up to the task of the transfer and concern that the patient's condition would get worse. Meanwhile, most of the family members expressed their willingness to trust the skilled transfer skills of the medical staff. Even as they worry, families tried to convince themselves that the transfer was a routine task.

Reexamination the day before yesterday said that the bleeding in the head had reduced, and I was relieved. In the evening, I wanted to go back to shower, but suddenly I got a call saying my husband was unresponsive. I did not know who to call for help (alas). (N2)

Well, there was some concern, and perhaps that's what I thought the first time, that they do this from time to time (transfer patients between CT rooms), so it must go well. (N6)

She (the nurse) would escort him along for tests and talk to us and it felt great that we were getting information, not enough, but I think she told me some and I was pleased about that. (N4)

#### 4.1.2 Sub‐theme 2: Complex internal activity during transit

- Negative experience under psychological crisis. (a) Grief and enthusiasm interweave: in the face of patients with disturbance of consciousness or even coma, most family members were out of control when they saw the patient, and were eager to wake up the patient and delay the time of transportation. (b) Coexistence of fear and anxiety: although it usually did not take a long time to complete, IHT can be a dangerous process for critically ill patients due to sudden changes in the treatment environment, monitoring or medical resources. These changes may make it difficult to identify important changes in a patient's condition or manage the occurrence of adverse events.

When my mother saw my father at the NICU door, she shouted for him, and my grandmother was crying in his hospital bed. The nurse was so anxious for us to push the bed back and forward quickly that we were all in a panic. (N3)

Before I knew it, a scene like that on TV was actually before my eyes, and the nurse suddenly noticed that

grandpa was breathless, and quickly climbed into bed and began to press on his heart, and I was so scared silly that my whole body shook, and it was so thrilling. (N6)

I am also a doctor and was wondering if the ventricular drain should be closed during the transfer. After all, there were still 10 minutes before and after the transfer, could this have affected the intracranial pressure fluctuation and caused a change in condition? That's what concerns me. (N1)

- Positive experience under psychological literacy: positive adjustment and full of hope. Conditions for critically ill neurological patients in emergency departments were changing rapidly, and their families were under great psychological stress. However, once the patient has made the transition to a period of stability, the emotions of the family members also returned to stability, cooperated highly with the medical staff during the transfer, adjust their psychology and began to hope that the disease would enter a new phase.

The first time to do the examination, that call a confusion, your heart ah, also be pulled, can not think properly, experienced a few times after ah, I can have experience, not the teacher said, we several family members have a good tacit understanding to hold my brother to (CT bed) up. (N4)

When I heard that the oedema in my son's head had almost subsided, my faith in not giving up came back. I thought being moved out of the ICU was a good thing for him and meant he was one step closer to recovery. (N7)

#### 4.1.3 Sub‐theme 3: Ambivalence after transfer

- Sense of urgency. Most family members wanted to know the results of relevant tests, and whether the patient was stable or post‐operative as soon as possible after being transported.

Every time I rang to ask about the results of the re‐examination, the nurse would say that the doctor would look at the film on the computer and if there was a problem, they would contact your family on their own initiative and you would just wait outside. Well, you really have no idea what a process this is, which means you will not know until tomorrow morning. (N10)

- Sense of helplessness. Intensive care was a difficult process for both patients and their families. In this process, the patient's critical condition and the family's responsibility for the patient's related decisions may have a negative impact on the patient's mental health, causing stress and anxiety. The closed management of the NICU prevented family members from following the patient's condition visually and dynamically after transport and increased the family's sense of isolation and helplessness.

My father had a ruptured blood vessel in his head, so I needed to decide immediately whether to have surgery. The situation was critical, and after my dad was transported here, I couldn't make up my mind. The doctors just explained the risks so clearly that I was left to worry outside the care unit. (N9)

I thought that my son transferred from ICU to NICU and the situation would be better, but the doctor placed a drainage tube in my son's lower back, and the door of the NICU cut off my contact with my son. Alas, also I did not know what was the matter inside. (N7)

### 4.2 Theme 2: Perception of problems in IHT

This theme mainly contained the following three sub‐themes: poor doctor–patient communication, weak awareness of risk assessment and deficiencies in the transfer procedure.

#### 4.2.1 Sub‐theme 1: Poor doctor–patient communication

Busy transport, difficulties in obtaining information and difficulties in absorbing information were considered to be the reasons affecting information transmission. Advance access to information suggested a sense of comfort and freedom from anxiety. Medical staff informed family members of the news of the transfer in a command tone, usually in an abrupt and urgent manner, which made it difficult for family members to adjust their psychological preparation and to know their coping strategies, which reduced their experience of the transfer to a certain extent.

I really hope that they (medical staff) can take time out of their busy schedule to explain the whole process of transport to us, and make a phone call to describe it simply, so that we would not be so scared. (N7)

I could not wait outside all the time, and I did not know what preparations to make beforehand. A doctor suddenly told me at 9am that my husband could be moved out and that a nurse would be in touch until 11am, but the nurse said the patient had not been discharged and my husband should be transferred in the afternoon. (N8)

#### 4.2.2 Sub‐theme 2: Weak awareness of risk assessment

There was a weak awareness of risk assessment and the quality of transport needed to be improved. In the study, some family members expressed the need for improved awareness of transportation safety for medical personnel.

- Neglecting the consideration of patient safety in transport attitude. Physical and human resources are both enablers (when available) and barriers (when lacking) to the transfer of quality care. Families reported inadequate preparations for the handover, with adequate human resources and necessary equipment not available.

I didn't think it's safe to have only nurses and junior doctors. Why didn't such a heavy patient be Accompanied by a senior doctor? (N7)

After pushing my dad out, the nurse ran outside the room to borrow oxygen cylinders. Wouldn't that delay transit time? My dad would be safe without monitoring in this situation? (N1)

- Lack of safety technology guarantee in transport operations. For patients with special conditions such as restlessness, the medical staff did not carry the appropriate medical articles in advance.

My father was very restless before the examination, and his whole body was restrained, and he was put on the bed (CT room). The inspector said that he had to inject some medicine, and then waited for a while for another nurse to send what medicine to come, and then my father was quiet. The waiting time before and after this was long, was there no hidden danger. (N3)

The sound of phlegm was heard incessantly. I saw that the nurse was extremely calm and never said anything, but said that when my brother returned to the NICU after the exam, he would be fine to have the sputum suctioned out. (N4)

#### 4.2.3 Sub‐theme 3: Deficiencies in the transfer procedure

This study highlighted some shortcomings of IHT, mainly reflected in the handover of both sides of the transport, the failure to effectively allocate medical resources and avoid potential risks in the transport. Most family members hoped to know the relevant examination results, whether the condition was stable or the patient's condition after surgery as soon as possible after transport.

- Lack of accurate docking time with the examination room. Communication and coordination in the run‐up to the transfer were extremely crucial. Contact the target area, formulate the transfer route, plan the transfer time and obtain cooperation after communication with family members to avoid adverse events caused by inadequate communication and preparation.

The nurse told me in a hurry that my husband was going to have an examination. After I called my family members to the CT room, the inspector said that there were too many emergency CT in this time period, and we needed to make a phone call to make an appointment in advance. At present, we could only wait in line! (N2)

- Lack of continuous communication with the transferred departments. After being discharged from NICU, under the one‐to‐numerous nursing model in the general ward, family members would have doubts about the quality of nursing service and worried about the reduction of attention to patients and nursing quality due to the limited number and ability of nursing staff in the ward and the heavy workload.

I must be glad to be transferred to the outside (general ward), which means improvement. My concern was whether the treatment inside was the same as the treatment outside. Do they had any details about my son? (N10)

The doctor said my grandfather needed an infusion of albumin to get rid of the edema in his head. The nurse asked me last night how many times the albumin had been given, and how many vials were left, and I do not think they had communicated the plan of treatment to each other. (N6)

### 4.3 Theme 3: Consciousness of the real needs

#### 4.3.1 Sub‐theme 1: Emotional respect and closeness

The empathy and friendly attitude of medical staff can bring great psychological comfort to the family members. Most family members claimed that they were able to stay in contact with the medical staff before and after the transfer, and that talking closely with the medical staff about the precautions for the transfer effectively soothed the negative emotions of the family members. Some families said that medical staff did not respect patients' wishes and were only anxious to complete their assigned tasks.

I remembered one incident that struck me. The nurse teacher was very skilled in telling us how to work with the family, how to try to get my son safely out of bed, and told me in detail how to push hard and where I could get film. My heart was very warm (smile). (N7)

I understand they look at transport it from the aspects of health care and logic, but we view it from the aspects of emotion, logic and medicine, please appropriate privacy, as soon as I opened the quilt see dad naked, as a family we see this scene in the mind is uncomfortable. (N1)

#### 4.3.2 Sub‐theme 2: Stay informed of the progression of the disease

Family members often supported the patient and acted as surrogate decision‐makers, preferring to understand as much information as possible, believing that even bad news was better than not knowing. Most family members have expressed an urgent desire to be kept informed of the latest test results, possible treatment options and prognosis so that coping strategies can be developed as soon as possible. Some family members also suggested that doctors commonly told themselves about patients' conditions in a condescending way, which increased the family's concerns about transportation to some extent.

What result must know, no matter good or bad, our mood is really very suffering. (N3)

No news is the best news, but as long as the doctor doesn't talk to us about the results of the follow‐up examination and the risks of the operation, the relatives at home will always keep guessing. (N6)

The doctor told us very directly that my husband's condition may not have a chance to improve, and the treatment plan in your department is the current one, and turning to the rehabilitation department to do functional exercise may help his recovery, but the right to decide is up to us. (N4)

#### 4.3.3 Sub‐theme 3: Greater social support

Social support has a positive effect on the individual's mental health, supports the individual's coping efforts and reduces social isolation. Most family members expressed a lack of emotional support from family members, as well as huge decision‐making risks and ongoing psychological distress during inpatient transfers. Some family members said they have been given great confidence by the encouragement of their patients' families, which was based on emotional resonance. Family members were always happy to help, and there was spiritual comfort.

Everyone has a home of their own. You cannot always call for help, especially since my mother's lung infection is so bad. She always needs to be re‐examined. (N5)

Life is at stake at any moment, and all matters of the head are not trifles. This matter must be decided by oneself. (N8)

I really appreciate the help of uncle Li. Every time my father does the examination, he is always very willing to help me, very enthusiastic to help me move my father to the bed in the examination room, and often comfort me, thank you, thank you, give me a lot of strength! (N9)

## 5 DISCUSSION

Family members of NICU patients experience a complex set of negative emotional experiences such as fear, anxiety, sadness, urgency and helplessness during IHT. Previous studies (de Grood et al., 2018; Karlsson et al., 2020) also confirmed this. The negative feelings of the family members are reinforced by the internal experiences of the family members at different stages and their perception of problems with IHTs, poor communication between medical staff, weak awareness of risk assessment and shortcomings in the transfer process. The root of this problem is that medical staff and family members had insufficient understanding of each other's roles, needs, expectations and even limitations (Li et al., 2023). These factors suggest that medical staff should thoroughly consider the psychological feelings of family members and understand the causes of family stress before transport.

Because ICU admission can occur suddenly, family members experience severe acute distress and do not have enough time to make accurate decisions and respond effectively (Avci & Ayaz‐Alkaya, 2022; Saeid et al., 2020, 2022), while facing the uncertainty and high risk of IHT NICU transport and being asked to make difficult alternative decisions in an unfamiliar and sometimes overwhelming environment (Harlan et al., 2020; Weber et al., 2021), therefore, may experience adverse psychological problems. Family members usually adopted six strategies to cope with these emotions, namely, problem solving, information seeking, avoidance, self‐reliance, support seeking and adaptation (Harlan et al., 2020). It is suggested that medical staff should be keen to capture family members' negative emotions during IHT and apply some intervention measures as soon as possible to reduce family members' psychological distress, which includes the use of diaries, training programs, decision AIDS or communication facilitation (Harlan et al., 2020). These strategies are designed to improve the active service awareness of medical staff, promote precautions in transfer in advance, convey disease information in time and stabilize the emotions of family members. At the same time, we should focus on optimizing the transfer process, preventing transfer risks, improving the quality of safe transfer and alleviating the anxiety of family members. On the other hand, family members are encouraged to boldly express their inner demands, and two‐way communication helped family members to repair their psychological problems, so as to improve their experience of IHT.

Neurosurgical transport adverse events can be divided into disease‐related and technology‐related adverse events (Zeng et al., 2021). IHT is a high‐risk process. Even if it only takes about 10 min, it may cause various physiological adverse consequences for the patient (de Grood et al., 2018). For critically ill patients in NICU, high‐quality IHT is essential for timely diagnosis and treatment and for reducing mortality (Bergman et al., 2020). Moreover, standardized transport behaviour is particularly important to effectively shorten the transport time and reduce the incidence of adverse events in transport (Yang et al., 2022). Therefore, the condition of patients should be carefully evaluated before transport and the risk of patient transport should be identified, which is directly related to the life safety of patients (An et al., 2022).

This study revealed that the lack of awareness of risk assessment by medical staff prior to transport was manifested first and foremost in the lack of consideration for patient safety in transport attitudes and the lack of safety technical guarantees in transport operations. Second, it highlighted shortcomings in the handover process and the need to standardize the management of the handover. The main manifestations were a lack of accurate timing with exam rooms and a lack of continuous communication with transferred departments. IHT of NICU patients was a continuation of the whole process of patient treatment and resuscitation. A high‐quality and efficient transfer plan and reasonable process would increase the trust and satisfaction of patients and their families.

A study (Bergman et al., 2020) aimed at improving the quality and safety of IHT of critically ill patients shows that emphasizing individual and collective non‐technical skills of healthcare workers, such as situation awareness and teamwork, is critical to ensuring that IHT safety hazards are predicted and corrected before patient harm is caused. Regarding risk prediction, a prospective cohort study (An et al., 2022) advocated the establishment of a simple scoring system that would enable nurses to quickly, conveniently and accurately identify patients whose condition may change during IHT. The application of near‐error management in IHT of critically ill patients can effectively manage the transport goods and personnel, comprehensively control the patient's condition, reduce the omission of items, shorten the duration of transport and eliminate the influence of external factors such as personnel and equipment based on the transfer checklist (Zhang et al., 2019).

Academics from home and abroad have also actively explored how to optimize the IHT process in NICUs. For example, a standardized, evidence‐based transport process covered the following aspects: make adequate preparations before transport, it is essential to evaluate whether the patient's condition is suitable for transport in advance, prepare the drugs and equipment needed to deal with unpredictable disease changes during transport, conduct effective communication and coordination with relevant departments before transport, formulate transport routes, contact family members for cooperation and establish a professional transport team with transport qualifications (Zeng et al., 2021). Some foreign scholars (Lu et al., 2022) have explored the role of Toyota Production System (TPS) in improving the quality management of emergency IHT of critically ill patients, and combined TPS management tools with PDCA methods. Positive changes in critical patient transit times, admissions, patient satisfaction and adverse event rates are confirmed. After the transfer is completed, the benefits and risks of transfer need to be evaluated again, such as accurate assessment of the disease, reasonable formation of the transfer team, predictive preparation of goods, effective communication, targeted measures and evaluation of whether to take the best transfer path, so as to provide a basis for continuous improvement of the transfer plan (Yue & Shi, 2019). It is suggested that medical staff should use scientific methods, accurately and timely to take effective measures to reduce the incidence of adverse events in NICU IHT, shorten the duration of transport, improve the quality of transport and further demonstrate the connotation of high‐quality nursing service, so as to improve the satisfaction of patients' families.

The theme of World Patient Safety Day 2023 is ‘Engaging Patients in Patient Safety’, recognizing the important role that patients, families and caregivers play in the safety of health care. Evidence (Avci & Ayaz‐Alkaya, 2022) shows that when patients are seen as partners in healthcare, significant improvements are achieved in terms of safety, patient satisfaction and health outcomes. Most Family members experience different psychological problems during the patient's ICU admission and hospitalization, currently referred to as family intensive care unit syndrome (FICUS) (Netzer & Sullivan, 2014). FICUS refers to a series of psychological problems that occur in family members as a result of the patient's ICU stay, which can severely affect the health of family members and lead to problems with rest, sleep and limited physical activity (Gertrude et al., 2019; Saeid et al., 2022). Social support is defined as resources or assistance provided by an individual or group that ensured successful coping, increased satisfaction and improves quality of life (Avci & Ayaz‐Alkaya, 2022).

The Clinical Practice Guideline for Patient‐Centred Family Support in the ICU (Davidson et al., 2017) suggests that communication, family involvement in care, training of family and ICU staff, provision of supportive resources, environmental factors and institutional processes should be considered when developing a family‐centred care program in the ICU. Family member satisfaction with ICU is positively correlated with multidimensional perceived social support and provided evidence that family education programs have been useful for ICU families and should be incorporated into clinical care (Avci & Ayaz‐Alkaya, 2022). Effective communication, good decision‐making, respect and compassion are key factors for patient and family satisfaction (Jo et al., 2019). In addition, supporting family members can help improve patient outcomes by enabling families to provide more effective care.

Therefore, it is necessary to understand the needs of family members in the whole process of IHT and build a family‐centred health education model according to the emotional experience corresponding to different stages of transfer, so as to realize the continuation of information exchange between the hospital and family. The family of the patient is often the forgotten part, and according to family‐centred care, the care plan should include both the patient and the family and start from the moment the patient is admitted to the hospital. Consideration should be given to the patient and family caregiver as one unit, acknowledging the role and expertise of the caregiver and including them in the care plan (Charles, 2020). First, FICUS screening by NICU nurses is necessary to provide a care plan for family members. Second, quality education of NICU staff about supporting these family members was essential, because when communication skills such as active listening and empathy are used to communicate with families, families feel more satisfied and are able to actively participate in the decision‐making process when clear and honest information is conveyed to families in understandable language. At the same time, we can also carry out joint and flexible meetings between medical staff and family members, timely introduce the current transfer plan and process, reveal the constraints of the existing resources and environment system and obtain the understanding of family members through frank communication, so as to alleviate the unhappy symptoms and experiences of family members during the IHT, build trust in medical staff, reduce conflicts and improve the quality of nursing.

## 6 LIMITATIONS

There are some limitations to our study. First, in the interview process, family members were affected by the disease and their emotional experiences and needs were mostly focused on the prognosis of the patient's disease, which was less relevant to the topic of this study and was not adequately presented. Second, the study subjects were from only one tertiary hospital in Hubei Province and the sample representation was limited. In the future, multidimensional and multicentre qualitative studies should be conducted for medical staff to further detect deficiencies in the IHT process in different departments and thus provide a reference for improving the quality and process of IHT.

## 7 CONCLUSION

Three themes emerge from interviews with family members of NICU inpatient transport patients: the inner experience of different stages of NICU inpatient transport, advice on perceiving the presence of inpatient transport and the inner support needs. The results show that the real experience of the patient's family during the IHT is complex with multiple support needs, suggesting a wide range of deficiencies in the transfer process, the transfer process needed to be further standardized. In the future, it is necessary to standardize the NICU IHT process training, build an intra‐hospital safe transport mode in which medical staff and family members participate together and meet the social support needs of family members, so as to improve the IHT experience and medical satisfaction of family members.

## AUTHOR CONTRIBUTIONS

Interview, data collection, data analysis and writing first draft preparation: Guo Xuan. Data collection, data analysis and proofreading: Liu Fei. Design, supervision and review of the whole project: Ding Juan. Revised version of the original manuscript, polished language: Zeng Xurui. All authors discussed results, provided feedback and confirmed and agreed with the content of the article.

## FUNDING INFORMATION

This work was supported by the Hubei Provincial Science and Technology Innovation Special Project (File Number: 2023‐026‐01).

## CONFLICT OF INTEREST STATEMENT

The authors declare no conflicts of interest.

## ETHICS STATEMENT

The study was approved by the ethical review committee of Jingzhou Hospital, which is affiliated with Yangtze University. Participants were notified verbally and in writing that their information would be kept confidential. They were also notified of their right to withdraw from the study at any time without providing a reason.

## Data Availability

Data supporting the findings of this study are available upon request from the first author. The data cannot be made public due to privacy or ethical constraints.
